# The Association of Blood Eosinophils and Neutrophils Expressing Eosinophilic Surface Markers with the Severity and Outcome of COVID-19

**DOI:** 10.3390/microorganisms12122503

**Published:** 2024-12-04

**Authors:** Jun Wang, Xin Li, Jiaqi Ren, Yafei Rao, Yixian Qiao, Lina Sun, Ying Liang, Chun Chang, Qingtao Zhou, Yongchang Sun

**Affiliations:** Department of Respiratory and Critical Care Medicine, Peking University Third Hospital, Research Center for Chronic Airway Diseases, Peking University Health Science Center, Beijing 100191, China; kobezijin24@163.com (J.W.);

**Keywords:** COVID-19, eosinophils, neutrophils, eosinophilic surface markers

## Abstract

(1) Background: The implication of type 2 (T2) inflammatory response in COVID-19 remains controversial. This study aimed to evaluate the association of eosinophils, neutrophils expressing eosinophilic surface markers and T2 cytokines with the severity and outcome of COVID-19. (2) Methods: Patients who were admitted to hospital due to COVID-19 from 18 December 2022 to 31 January 2023 were enrolled. Peripheral blood WBC and differentials, T2 cellular markers (subsets of eosinophils and neutrophils expressing eosinophilic surface markers) and cytokines at admission were measured and compared between subjects with different disease severities and outcomes. (3) Results: Ten mild-to-moderate and 22 severe-to-very severe cases were enrolled for analysis. Of these patients, seven died of severe-to-very severe disease. The severe-to-very severe patients showed a higher number of neutrophils, but lower numbers of eosinophils, lymphocytes cells and neutrophils expressing eosinophilic surface markers. Similarly, deceased cases were also characterized by increased neutrophils, but decreased eosinophils and neutrophils expressing eosinophilic surface markers. The levels of T2 cytokines failed to demonstrate a significant correlation with the severity or outcome of COVID-19. (4) Conclusions: Eosinophils and neutrophils expressing eosinophilic surface markers were associated with milder disease and better outcomes of COVID-19, suggesting that a T2 inflammatory response may confer a potential protective effect against the disease.

## 1. Introduction

It is widely recognized that COVID-19 has the potential to alter the profile of inflammatory cells, potentially triggering a cytokine storm. A hallmark of the inflammatory response in COVID-19 is characterized by an elevation in neutrophils, and a reduction in eosinophils and lymphocytes [1,2]. These fluctuations in cell populations may serve as predictive indicators for mortality rates [3] and the severity of the disease in COVID-19 patients [4,5]. 

Eosinophils have emerged as potential biomarkers in respiratory viral infections [6]. Notably, a depletion in eosinophils, referred to as eosinopenia, could signal a dire prognosis of COVID-19 [7,8]. The severity of eosinopenia worsening over time may indicate a progression to a critical phase of COVID-19, along with an escalated risk of mortality [9,10,11,12]. However, pulmonary eosinophilia has not been identified as a component of the pulmonary pathology associated with *SARS-CoV-2* to date [13]. The precise role and impact of peripheral blood inflammatory cells, particularly eosinophils, in the context of COVID-19 warrant further investigation. 

The distinct severity of COVID-19 in patents with COPD or asthma hinted that the severity of COVID-19 could be modulated by type 2 inflammation [14]. It is interesting to note that asthma patients characterized by low Th2 inflammation face an elevated risk of testing positive for *SARS-CoV-2* [15], developing severe COVID-19 [15,16] and having adverse outcomes from COVID-19 [15,17]. Th2-driven inflammation is crucial for the initial defense against viral incursions [18,19], and appears to be associated with reduced disease severity [19] and mortality post-infection [20]. Specific cytokines associated with a T2 immune response may confer a potentially protective mechanism against the ravages of COVID-19 [21,22,23]. However, this protective potential of T2-high inflammation was not mirrored in patients with severe COPD [24]. Furthermore, there is conflicting evidence suggesting that allergic conditions have been linked to a higher risk of COVID-19 diagnosis [20], and Th2-driven inflammation contributes to the late-phase hyperinflammation observed in the severe stages of COVID-19 [18] and is correlated with severe COVID-19 [25]. Consequently, the intricate dynamics of a T2 inflammatory response in COVID-19 necessitates further investigation to fully uncover the underlying biological mechanisms.

Eosinophils and neutrophils are heterogeneous cells with potentially multiple subsets in health and disease [26]. It is intriguing to note that eosinophil-specific cell surface markers such as IL-5Rα (CD125) [27,28,29,30], Siglec-F (Siglec-8) [31,32] and CCR3 (CD193) [33,34,35,36], have been reported to express on the surface of neutrophils. Immature metamyelocyte neutrophils in humans expand during severe asthmatic inflammation and express both neutrophilic and eosinophilic markers [37]. Whether these unique subsets of neutrophils (expressing eosinophilic surface markers) are disturbed in COVID-19 remains unexplored.

## 2. Materials and Methods

### 2.1. Study Design

This was a prospective cohort study performed at the Peking University Third Hospital. Thirty-two patients who were admitted to hospital due to COVID-19 during the outbreak of COVID-19 from 18 December 2022 to 31 January 2023 were enrolled in our study, each fulfilling the diagnostic criteria for COVID-19 outlined in the Tenth Edition of China’s COVID-19 Guideline, and according to which the patients were categorized into two severity groups: a mild-to-moderate group and a severe-to-very severe group, comprising 10 and 22 individuals, respectively. Blood samples were collected at admission, and the WBC count and differentials, subsets of eosinophils and neutrophils expressing eosinophilic surface markers, and cytokine levels were measured. The differences in inflammatory cells, T2 cell markers and cytokines were analyzed between subjects with different COVID-19 severities and outcomes.

The study was approved by the Ethics Committee of Peking University Third Hospital (approval code: IRB00006761-M2022865). All the procedures were performed in accordance with the guidelines of the authors’ institutional ethics committee and adhered to the tenets of the Declaration of Helsinki.

### 2.2. Clinical Data Collection

Demographic data, onset days, comorbidity, treatments (i.e., respiratory support, ICU admission, corticosteroid therapy), laboratory findings, length of stay and in-hospital mortality were collected for the 32 patients. The date of disease onset was defined as the day when symptoms first appeared, including fever, shortness of breath, dyspnea, cough and/or expectoration, headache, chest pain and pharyngalgia.

### 2.3. Blood Sample Collection, Processing, and Isolation of White Blood Cells

Peripheral venous blood (4 mL) was collected and stored in ethylenediaminetetraacetic acid (EDTA) in anti-coagulant tubes. Samples were centrifuged at 500× *g* for 10 min at 4 °C. The plasma was used for detecting cytokines, and the precipitated blood cells underwent two rounds of red blood cell lysis and centrifugation to obtain precipitated white blood cells. Cells were harvested and washed twice with PBS at 500× *g* for 5 min and prepared for flow cytometry.

### 2.4. Flow Cytometry

Cells (1 × 10^6^) from peripheral blood were stained with indicated monoclonal antibodies (mAbs) for 15 min in the dark under ice incubation conditions, and were fixed with 1% paraformaldehyde. Flow cytometric analysis was performed on CytoFLEX S (Beckman Coulter, Brea, CA, USA). Data were analyzed by using Cytoexpert v. 2.3 software. All antibodies used are listed in Supplementary Table S1.

The eosinophilic surface markers analyzed included CD193 [38,39,40,41], Siglec-8 [42,43,44,45] and CD125 [46]. To ensure the accuracy and reliability of the gating strategy, one-point reduction control was implemented when setting the thresholds for each gate. This meticulous approach guaranteed that our analysis reflected the true cellular profiles, contributing to the validity of our findings.

In conducting the flow cytometry analysis, the forward scatter area (FSA) and forward scatter height (FCA) were employed to morphologically and densitometrically identify the granulocyte population. CD45 and CD15 were utilized to distinguish granulocytes and neutrophils, respectively. Granulocytes were categorized into three populations based on their expression levels of CD15 and CD45: CD15+CD45mid, CD15+CD45high and CD15−CD45high. The CD15+CD45high and CD15−CD45high populations expressing eosinophilic surface markers were classified, respectively, as neutrophils expressing eosinophilic surface markers and classical eosinophils. In contrast, the CD15+CD45mid population, which lacked eosinophilic surface markers, was classified as classical neutrophils. Furthermore, we examined the expression of three eosinophilic markers within the CD15+CD45high and CD15−CD45high populations, resulting in the delineation of six distinct cell subpopulations, i.e., the CD15+Siglec-8+, CD15+CD193+, CD15+CD125+, CD15−Siglec-8+, CD15−CD193+ and CD15−CD125+ subsets (Figure S1).

### 2.5. Cytokine Profiling by LEGENDplexTM

The concentrations of 12 cytokines and cytotoxic molecules were analyzed in plasma (n = 32) using the LEGENDplexTM Human Th cytokine Panel (12-plex, BioLegend, San Diego, CA, USA). The assay was performed according to the manufacturer’s instructions. Flow cytometric analysis was performed on CytoFLEX S (Beckman Coulter). Data were analyzed using CytExpert 2.5 and online software (BioLegend, website: https://legendplex.qognit.com/user/login?next=home, 2 September 2024).

### 2.6. Statistical Analysis

Data were expressed as the mean ± standard deviation or median (interquartile range, IQR) for continuous variables depending on whether or not they followed a normal distribution, while categorical variables were expressed as counts and percentages. In order to meet the normal distribution, some data underwent natural logarithmic transformation before making *t*-test. Missing values were not imputed. All reported probability values were two-tailed, and a *p*-value less than 0.05 was considered statistically significant. Statistical testing included the *t*-test (data conformed to the normal distribution), Mann–Whitney U-test (data not conformed to the normal distribution), Chi square and Fisher’s exact tests. GraphPad Prism 9.5.1 and SPSS 20 were used for the graphic representation and statistical analysis.

## 3. Results

### 3.1. Demographic, Clinical and Prognosis Characteristics

Of the 32 cases of COVID-19, 10 were classified as mild-to-moderate, while 22 were severe-to-very severe, of which 7 died during hospitalization. The demographic and clinical characteristics of the patients are shown in Table 1. The severe-to-very severe cases had a higher rate of ventilator use, intensive care unit admission, corticosteroid therapy and mortality, and a lower oxygenation index compared with the mild-to-moderate cases (Table 1).

### 3.2. Increased Blood Neutrophils but Decreased Eosinophils and Lymphocytes in Patients with Severe-to-Very Severe Disease

The blood WBC differentials and inflammatory markers are shown in Table 2. The severe-to-very severe cases exhibited higher neutrophils, but lower eosinophils and lymphocytes.

### 3.3. Decreased CD125+ Eosinophils in Patients with Severe-to-Very Severe Disease

**Figure 1:**
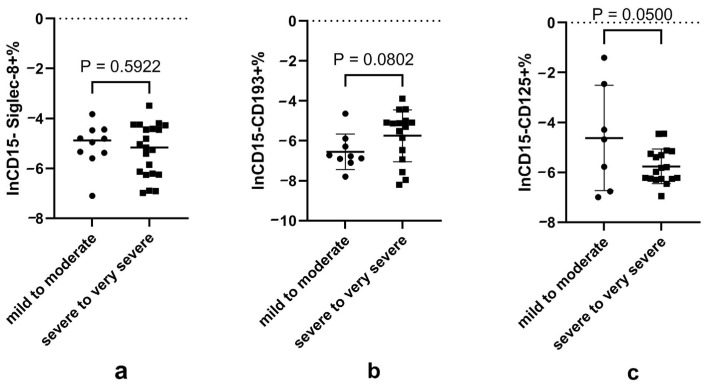
Proportion of eosinophil subsets according to disease severity. Notes: (**a**), (**b**) and (**c**) represents the natural logarithmic of the CD15−Siglec-8+, CD15−CD193+ and CD15−CD125+ proportion of granulocytes according to disease severity, respectively. Data did not conform to normal distribution and were analyzed using the *t*-test after natural logarithmic transformation; *p* < 0.05 is considered significant.

Given that blood eosinophils are reduced in severe-to-very severe cases and that eosinophils are heterogeneous as defined by surface markers and functions, we further explored whether there was any disturbance in the subpopulations of these cells. Our results showed lower CD15−CD125+ eosinophils, but numerically (but not significantly) higher CD15−CD193+ eosinophils in the severe-to-very severe cases compared to the mild-to-moderate group. Eosinophils expressing different surface markers in all subjects with different disease severities are shown in Table S2 of Figure 1.

### 3.4. Disturbed Neutrophil Subsets Expressing Eosinophilic Surface Markers in Patients with Severe-to-Very Severe Disease

**Figure 2:**
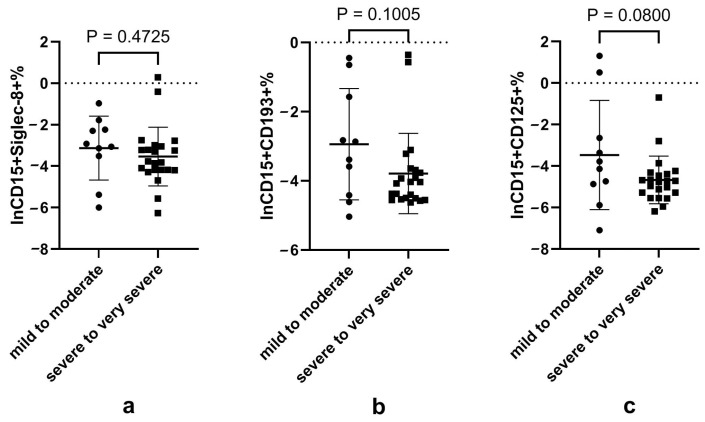
Proportion of neutrophil subsets expressing eosinophilic surface markers according to disease severity. Notes: (**a**), (**b**) and (**c**) represents the natural logarithmic of the CD15+Siglec-8+, CD15+CD193+ and CD15+CD125+ proportion of granulocytes according to disease severity, respectively. Data did not conform to normal distribution and were analyzed using the *t*-test after natural logarithmic transformation; *p* < 0.05 is considered significant.

Considering that blood neutrophils and eosinophils presented a contrary trend in the process of COVID-19, and that neutrophils have subsets expressing eosinophilic surface markers, we wondered if there was disturbance in the neutrophil subsets expressing eosinophilic surface markers and if this disturbance was associated with COVID-19 severity. The results showed that CD15+CD125+ neutrophils tended to be decreased in the severe-to-very severe case compared with the mild-to-moderate group (Table S2, Figure 2).

### 3.5. Blood Cytokines in COVID-19 Patients with Different Disease Severities

The concentration of peripheral blood cytokines for all subjects are presented in Table 3. No significant differences were observed in the cytokines measured. However, the concentrations of IL-6, IL-9, IL-17A, IL-4 and IL-22 demonstrated a significant correlation with eosinophils and neutrophils expressing eosinophilic surface markers (Figure S2).

### 3.6. Disturbed Eosinophils and Neutrophils, and Their Subsets in Patients That Died of COVID-19

**Figure 3:**
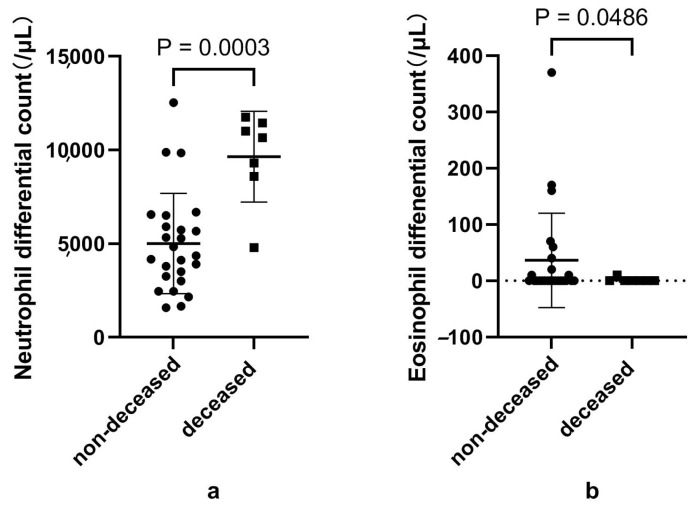
Differential count of neutrophils and eosinophils according to disease outcome. Notes: (**a**) and (**b**) represents the differential count of neutrophils and eosinophils according to disease outcome, respectively. Data were analyzed using the *t*-test; *p* < 0.05 is considered significant.

**Figure 4:**
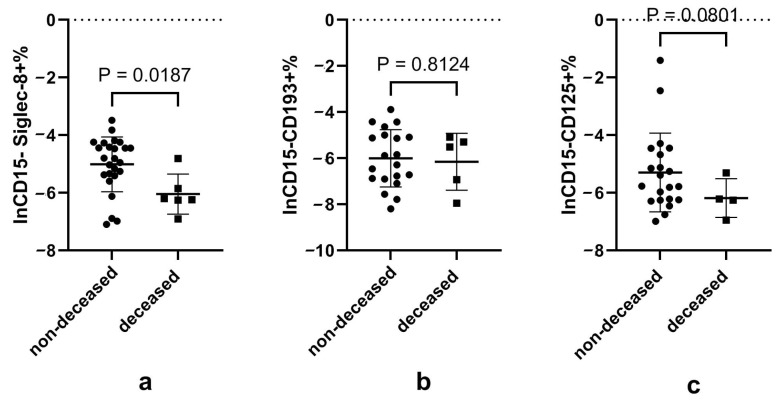
Proportion of different eosinophil subsets according to disease outcome. Notes: (**a**), (**b**) and (**c**) represents the natural logarithmic of the CD15−Siglec-8+, CD15−CD193+ and CD15−CD125+ proportion of granulocytes according to disease outcome, respectively. Data did not conform to normal distribution and were analyzed using the *t*-test after natural logarithmic transformation; *p* < 0.05 is considered significant.

When comparing patients who were discharged alive and those that died (Table S3) during hospitalization, we found higher neutrophils and lower eosinophils in the deceased patients (Figure 3). The deceased subjects also presented significantly lower Siglec-8+ eosinophils, while CD125+ eosinophils also showed a similar trend. The data of eosinophil subsets in patients with different outcomes are shown in Table S4 of Figure 4.

**Figure 5:**
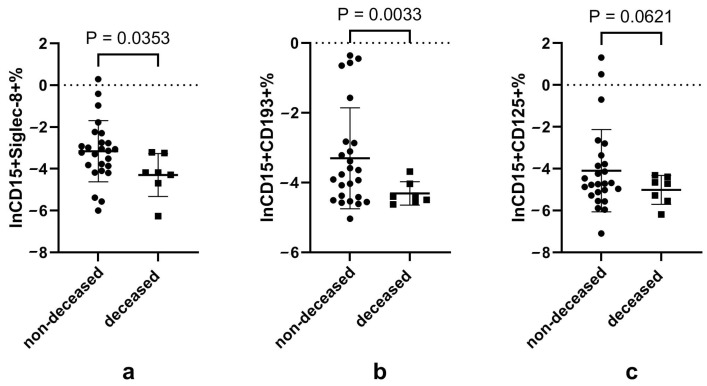
Proportion of neutrophils subsets expressing eosinophilic surface markers according to disease outcome. Notes: (**a**), (**b**) and (**c**) represents the natural logarithmic of the CD15+Siglec-8+, CD15+CD193+ and CD15+CD125+ proportion of granulocyte according to disease outcome, respectively. Data did not conform to normal distribution and were analyzed using the *t*-test after natural logarithmic transformation; *p* < 0.05 is considered significant.

Regarding the neutrophil subsets expressing eosinophilic markers, it was interesting to note a lower proportion of CD15+Siglec-8+, CD15+CD193+ and CD15+CD125+ neutrophils in the deceased subjects compared to those discharged alive. The proportions of neutrophils expressing eosinophilic surface markers in cases of different outcomes are shown in Table S4 of Figure 5.

The cytokine concentrations for all subjects and participants with different outcomes are presented in Table S5. No significant differences in peripheral blood cytokines were exhibited among patients with different outcomes.

## 4. Discussion

Consistent with previous reports [1,2,3,4,5,47], we found a decrease in blood eosinophils, together with an increase in neutrophils in patients with severe-to-very severe COVID-19. What is new to our study is that we found disturbed eosinophil subsets and neutrophils expressing eosinophilic markers in these patients, and this disturbance was associated with disease severity and hospitalization outcome, which provides a new insight for further exploring the potential role of eosinophils and T2 inflammation in the process of COVID-19.

Accumulating evidence points to the role of T2 inflammation in the susceptibility to, and progression and prognosis of COVID-19. However, there is still controversy on the protective or noxious effect of T2 inflammation in this disease. Our results indicate that eosinophils, the effector cells in T2 response, may have a protective role in the progression and outcome of patients with COVID-19, as suggested by the decreased eosinophil and neutrophil subsets expressing eosinophilic surface markers in severe and deceased cases. At the same time, the relationship between eosinophilic subsets and various T2 cytokines also enhances the reliability of our results.

Eosinopenia indicates more severe disease and a worse prognosis in COVID-19 [9,10,11,12]. Our results also presented a similar trend. Furthermore, we showed that the decrease in CD125+ and siglec-8+ eosinophils, and the increase in CD193+ eosinophils may be related to a worse severity and prognosis of the disease. There was a study showing that eosinophil-independent IL-5 levels were increased in critically ill COVID-19 patients who survived [23], and the same trend was present in our results. Siglec-8 is an apoptosis receptor and CD193 is a chemotactic receptor on the surface of eosinophils, which may suggest that as the disease progresses, the corresponding functions of apoptosis and chemotacsis were decreased and increased, respectively. The increase in CD193+ eosinophil-associated chemotactic function was also consistent with the inflammatory storm during the COVID-19 process.

Our study was the first to explore the proportions of the neutrophil subsets expressing eosinophilic surface markers. As characteristic surface markers of eosinophils, CD193, Siglec-8 and CD125 may be expressed on the surface of some neutrophils simultaneously. Our results suggest that these specific neutrophil subsets may participate in T2 inflammation and play a protective role in the process of COVID-19 through the expression of eosinophil surface markers.

As is well known, IL-5 signaling can stimulate neutrophil-dependent responses during respiratory viral infections via the CD125 (IL-5Rα) expressed on migrated neutrophils [27]. The inhibition of IL-5 can affect the virus resistance of COVID-19 patients [48], but it can also reduce hyperinflammation in ARDS models [49]. Our result, here, suggests the possible protective impact of CD125 expressed by neutrophils on the process of COVID-19. Alongside that, the expression of CD193 and siglec-8 by neutrophils may also have a similar effect.

The Siglec-F+ neutrophil subset was reported in an allergic rhinitis model [32], while the CD193+ neutrophil subset was detected in mouse models of influenza infection [33] and in infiltrated neutrophils from patients with chronic inflammatory lung diseases [34].

It is worth noting that hypercytokinemia is tightly linked to the disease severity and mortality rates of COVID-19 [3,25,50,51,52,53]. Although no significant differences were observed in cytokines and inflammatory factors between patients with different severities or outcomes in our research, their correlation with different eosinophilic subtypes still presented suggestive significances. There may be differences in the onset time between individuals, as well as variations in the use of glucocorticoids by patients, which may affect the levels of inflammatory factors [54].

Our study has several limitations. Firstly, the sample size was small, and therefore, there was an uneven distribution across varying degrees of disease severity. Nonetheless, we meticulously accounted for these disparities in our analytical and statistical approach. Furthermore, our examination of eosinophil phenotypes was limited as we were unable to perform a morphological analysis of the distinct cell populations. This aspect of the study calls for a more sophisticated experimental design in future research endeavors to facilitate a more extensive and profound exploration of these cellular characteristics.

## 5. Conclusions

In a cohort of COVID-19 patients with varying disease severities and outcomes, we found disturbed eosinophil subsets and neutrophil subsets expressing eosinophilic markers associated with disease severity and mortality. Our results provide a new insight for further understanding the potential role and mechanisms of eosinophils and T2 inflammation in the pathogenesis of COVID-19.

## Figures and Tables

**Table 1:** Demographic, clinical and prognosis characteristics of the patients with COVID-19.

	Mild-to-Moderate n = 10	Severe-to-Very Severe n = 22	*p*-Value
Age (mean ± SD, years)	67.3 ± 11.0	74.6 ± 13.1	0.139
Sex (male, %)	6 (60)	15 (68.2)	0.703
BMI (mean ± SD, kg/m^2^)	24.2 ± 3.2	23.7 ± 3.4	0.714
Comorbidity(n., %)			
Hypertension	8 (80)	14 (63.6)	0.440
Diabetes	4 (40.0)	5 (22.7)	0.407
Coronary heart disease	2 (20)	2 (9.1)	0.572
COPD	1 (10)	3 (13.6)	1
Asthma	1 (10)	2 (9.1)	1
Interstitial lung diseases	0 (0)	3 (13.6)	0.534
Onset days (mean ± SD, days)	8.4 ± 2.5	10.7 ± 2.5	0.27
Oxygenation index (mean ± SD, mmHg)	431 ± 129	199 ± 109	<0.001
Invasive ventilation (n., %)	0 (0)	3 (13.6)	0.534
Non-invasive ventilation (n., %)	0 (0)	11 (50)	0.006
ICU admission (n., %)	0 (0)	4 (18.2)	0.071
Length of stay (mean ± SD, days)	8.2 ± 1.9	16.4 ± 15.3	0.022
System corticosteroid therapy (n., %)	3 (30.0)	17 (77.3)	0.018
Mortality(n., %)	0 (0)	7 (31.8)	0.07

Notes: BMI: body mass index; ICU: intensive care unit; COPD: chronic obstructive pulmonary disease; SD: standard deviation. The numerical and categorical variables were analyzed using the *t*-test and Chi square test, respectively; *p* < 0.05 is considered significant.

**Table 2:** Blood WBC differentials and inflammatory markers of the patients with COVID-19.

	Mild-to-Moderate n = 10	Severe-to-Very Severe n = 22	*p*-Value
Neutrophils			
Differential count (mean ± SD, /μL)	4100 ± 1700	6900 ± 3400	0.018
Proportion (%)	70.3 ± 15.0	85.6 ± 6.0	<0.001
Eosinophils			
Differential count (mean ± SD, /μL)	90 ± 120	2 ± 5	0.001
Proportion (%)	1.50 ± 1.90	0.03 ± 0.11	0.001
Lymphocytes			
Differential count (mean ± SD, /μL)	1200 ± 600	600 ± 300	0.002
Proportion (%)	21.8 ± 12.5	9.49 ± 5.05	<0.001
PCT (mean ± SD, ng/mL)	0.56 ± 1.22	0.24 ± 0.24	0.234
CRP (mean ± SD, mg/dL)	7.0 ± 5.8	9.5 ± 9.4	0.473
D-dimer (mean ± SD, μg/mL)	0.4 ± 0.3	9.9 ± 35.7	0.411

Notes: SD: standard deviation; PCT: procalcitonin; CRP: C reactive protein. Data were analyzed using the *t*-test; *p* < 0.05 is considered significant.

**Table 3:** Peripheral blood cytokines in patients with COVID-19 according to disease severity.

	Mild-to-Moderate n = 10	Severe-and-Very Severe n = 22	*p*-Value
IL5 (median, IQR, pg/mL)	0.7 (10.6)	1.1 (2.6)	0.826
IL13 (median, IQR, pg/mL)	6.9 (10.6)	2.1 (18.0)	0.366
IL2 (median, IQR, pg/mL)	1.0 (3.6)	0 (1.9)	0.345
IL6 (median, IQR, pg/mL)	10.1 (30.0)	5.7 (30.0)	0.952
IL9 (median, IQR, pg/mL)	1.3 (10.2)	5.6 (10.4)	0.483
IL10 (median, IQR, pg/mL)	6.3 (7.1)	4.6 (8.3)	0.675
INFγ (median, IQR, pg/mL)	26.8 (34.4)	12.6 (42.5)	0.305
TNFα (median, IQR, pg/mL)	0.5 (81.8)	0.1 (20.2)	0.704
IL17A (median, IQR, pg/mL)	0.9 (3.6)	0 (2.1)	0.434
IL17F (median, IQR, pg/mL)	1.1 (8.4)	0 (1.4)	0.163
IL4 (median, IQR, pg/mL)	2.6 (5.3)	0.8 (3.0)	0.219
IL22 (median, IQR, pg/mL)	3.1 (10.9)	2.2 (7.6)	0.795

Notes: data did not conform to normal distribution and were analyzed using the Mann–Whitney U-test; *p* < 0.05 is considered significant.

## Data Availability

Data are contained within the article or Supplementary Material.

## Supplementary Materials

The following supporting information can be downloaded at: https://www.mdpi.com/article/10.3390/microorganisms12122503/s1, Supplementary Material 1: Table S1: Reagent used in this research; Figure S1: Representative gating strategy of subsets of eosinophils and neutrophils expressing eosinophilic surface markers; Table S2: Proportion of subsets of eosinophils and neutrophils expressing eosinophilic surface markers according to different disease severities; Figure S2: The correlation between subsets of eosinophils and neutrophils expressing eosinophilic surface markers with peripheral blood cytokines; Table S3: Demographic and clinical characteristic comparison in patients with different outcomes; Table S4: Proportion of subsets of eosinophils and neutrophils expressing eosinophilic surface markers in patients with different outcomes; Table S5 Peripheral blood cytokines in patients with COVID-19 according to disease outcome; Supplementary Material 2: Raw data of COVID-19 patients: raw data of the study.
