# Supplementary material for: The Association of Blood Eosinophils and Neutrophils Expressing Eosinophilic Surface Markers with the Severity and Outcome of COVID-19
Source: Microorganisms. 2024 Dec 4;12(12):2503. doi: 10.3390/microorganisms12122503 (PMC11727756; doi:10.3390/microorganisms12122503)
Supplement: Supplementary file 1 [file microorganisms-12-02503-s001.zip › Supplementary Material 1.pdf]

Table S1 Reagent used in this research

| Reagent | Manufacturer | Catalog | Clone |
|---|---|---|---|
| APC- Fire 750 anti-human CD45 | Biolegend | 368517 | 2D1 |
| BV 605 anti-human CD15 | Biolegend | 323031 | W6D3 |
| PE anti-human CD125 | BD PMG | 555902 | A14 |
| BV421 anti-human CD193 | Biolegend | 310714 | 5EB |
| APC anti-human Siglec-8 | Biolegend | 347106 | 7C9 |
| APC-Cy7 Zombie Aqua Fixable Viability Kit | Biolegend | 423101 | - |
| LEGENDplex™ Human Th cytokine Panel(12-plex) | Biolegend | 741028 | - |
| Fixation Buffer | Biolegend | 420801 | - |

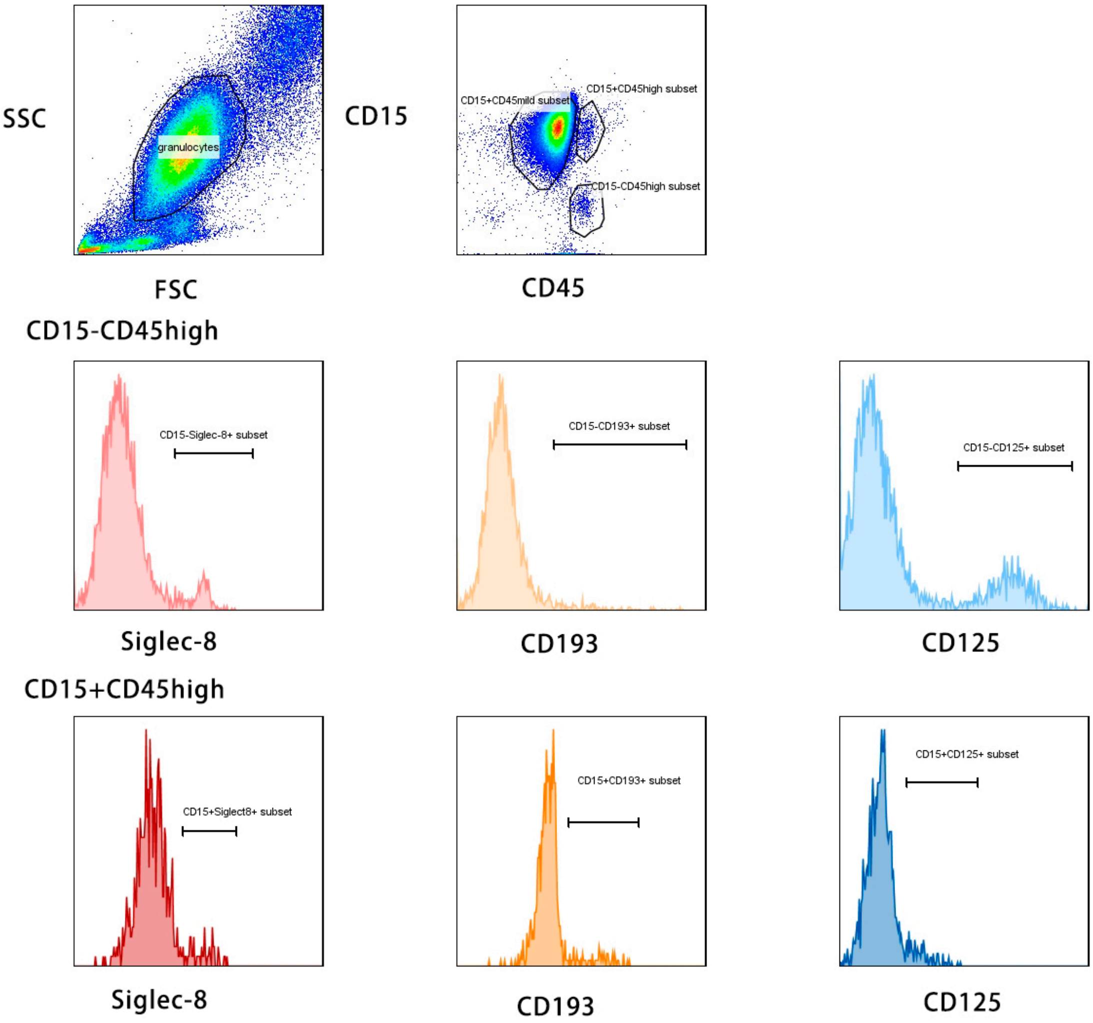


Figure S1 Representative gating strategy of subsets of eosinophils and neutrophils expressing eosinophilic surface markers

Notes: Granulocytes were categorized into three groups based on their expression levels of CD15 and CD45: $CD15^{+}CD45^{mid}$, $CD15^{+}CD45^{high}$, and $CD15^{-}CD45^{high}$ subsets; $CD15^{+}CD45^{high}$ population are categorized into three subsets: $CD15^{+}$Siglec-$8^{+}$, $CD15^{+}CD193^{+}$, $CD15^{+}CD125^{+}$; $CD15^{-}CD45^{high}$ population are categorized into three subsets: $CD15^{-}$Siglec-$8^{+}$, $CD15^{-}CD193^{+}$, and $CD15^{-}CD125^{+}$.

Table S2 Proportion of subsets of eosinophils and neutrophils expressing eosinophilic surface markers according to different disease severity

| | Total n=32 (median, IQR) | Mild to moderate n=10 (median, IQR) | Severe to very severe n=22 (median, IQR) |
|---|---|---|---|
| CD15- Siglec-8+%(%) | 0.006(0.01) | 0.008(0.01) | 0.006(0.01) |
| $CD15^{-}CD193^{+}$%(%) | 0.001(0.01) | 0.001(0) | 0.002(0.01) |
| $CD15^{-}CD125^{+}$%(%) | 0.002(0) | 0.002(0.03) | 0.002(0) |
| $CD15^{+}$Siglec-$8^{+}$%(%) | 0.038(0.05) | 0.051(0.1) | 0.023(0.03) |
| $CD15^{+}CD193^{+}$%(%) | 0.019(0.03) | 0.045(0.27) | 0.017(0.01) |
| $CD15^{+}CD125^{+}$%(%) | 0.009(0.01) | 0.019(0.46) | 0.009(0.01) |

Notes: CD15- Siglec-8+%: CD15- Siglec-8+ proportion of granulocyte; $CD15^{-}CD193^{+}$%: $CD15^{-}CD193^{+}$ proportion of granulocyte; $CD15^{-}CD125^{+}$%: $CD15^{-}CD125^{+}$ proportion of granulocyte; $CD15^{+}$Siglec-$8^{+}$%: $CD15^{+}$Siglec-$8^{+}$ proportion of granulocyte; $CD15^{+}CD193^{+}$%: $CD15^{+}CD193^{+}$ proportion of granulocyte; $CD15^{+}CD125^{+}$%: $CD15^{+}CD125^{+}$ proportion of granulocyte; IQR: interquartile range.

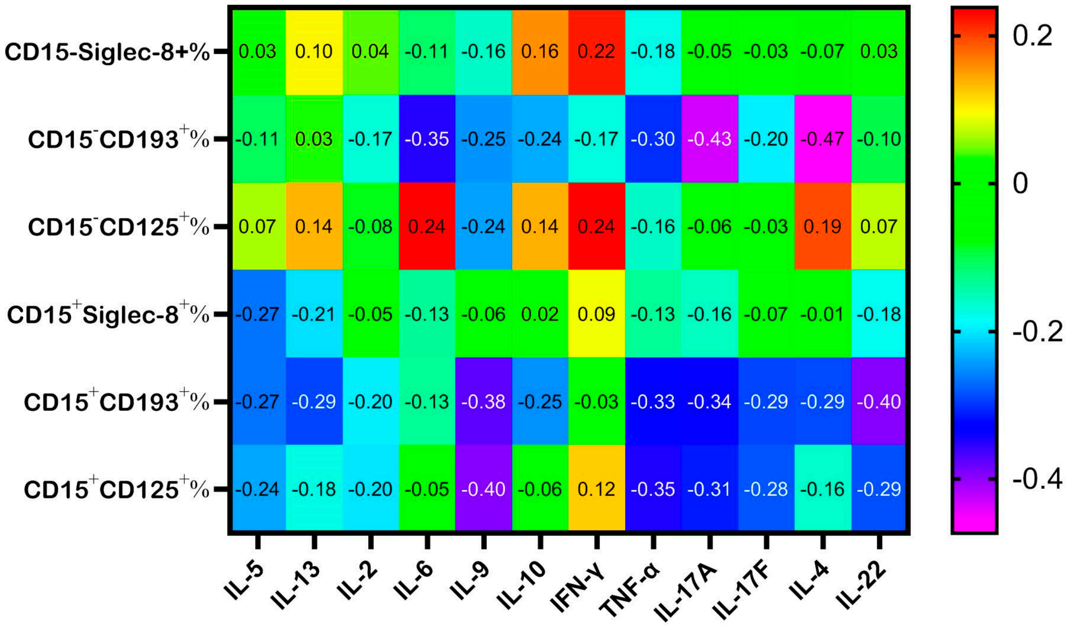

Figure S2 The correlation between subsets of eosinophils and neutrophils expressing eosinophilic surface markers with peripheral blood cytokines

Notes: CD15- Siglec-8+%: CD15- Siglec-8+ proportion of granulocyte; $CD15^-CD193^+$%: $CD15^-CD193^+$ proportion of granulocyte; $CD15^-CD125^+$%: $CD15^-CD125^+$ proportion of granulocyte; $CD15^+$Siglec-$8^+$%: $CD15^+$Siglec-$8^+$ proportion of granulocyte; $CD15^+CD193^+$%: $CD15^+CD193^+$ proportion of granulocyte; $CD15^+CD125^+$%: $CD15^+CD125^+$ proportion of granulocyte; each variable underwent natural logarithmic transformation before performing Pearson correlation.

Table S3 Demographic, clinical characteristics comparison in patients with different outcome

| | Total n=32 | Non-deceased n=25 | Deceased n=7 | *P* value |
|---|---|---|---|---|
| Age (mean ± SD, years) | 72.3 ± 12.8 | 69.8 ± 12.1 | 81.5 ± 11.7 | 0.030 |
| Sex (male, %) | 21 (67.7) | 16 (64) | 5 (71.4) | 1 |
| BMI (mean ± SD, kg/$m^2$) | 23.9 ± 3.3 | 23.9 ± 3.2 | 23.8 ± 4.1 | 0.971 |
| Comorbidity(n., %) | | | | |
| Hypertension | 22 (68.8) | 17 (68) | 5 (71.4) | 1 |
| Diabetes | 9 (28.1) | 7 (28) | 2 (28.6) | 1 |
| Coronary heart disease | 4 (12.5) | 3 (12) | 1 (14.3) | 1 |
| COPD | 4 (12.5) | 3 (12) | 1 (14.3) | 1 |
| Asthma | 3 (9.4) | 1 (4) | 2 (28.6) | 0.113 |
| Interstitial lung diseases | 3 (9.4) | 2 (8) | 1 (14.3) | 0.536 |
| Onset days (mean±SD, days) | 10.0 ± 5.3 | 10.4 ± 5.9 | 8.3 ± 2.0 | 0.302 |
| Oxygenation index (mean±SD, mmHg) | 267 ± 156 | 295 ± 164 | 170 ± 64 | 0.006 |
| Invasive ventilation (n., %) | 3 (9.4) | 2 (8) | 1 (14.3) | 0.536 |
| Non-invasive ventilation (n., %) | 11 (34.4) | 5 (20) | 6 (85.7) | 0.003 |
| ICU admission (n., %) | 4 (12.5) | 2 (8) | 2 (28.6) | 0.201 |
| Length of stay (mean±SD, days) | 13.8 ± 13.2 | 12.5 ± 10.2 | 18.4 ± 21.2 | 0.302 |
| System corticosteroid therapy (n., %) | 20 (62.5) | 15 (60.0) | 5 (71.4) | 0.683 |

Notes: BMI: body mass index; ICU: intensive care unit; COPD: chronic obstructive pulmonary disease; SD: standard deviation; The numerical and categorical variables were analyzed using t-test and Chi square test respectively, $P < 0.05$ is considered significant.

Table S4 Proportion of subsets of eosinophils and neutrophils expressing eosinophilic surface markers in patients with different outcome

| | Total n=32 (median, IQR) | deceased n=7 (median, IQR) | non-deceased n=25 (median, IQR) |
|---|---|---|---|
| CD15- Siglec-8+%(%) | 0.006(0.01) | 0.002(0) | 0.008(0.01) |
| $CD15^-CD193^+$%(%) | 0.001(0.01) | 0.001(0) | 0.001(0.01) |
| $CD15^-CD125^+$%(%) | 0.002(0) | 0.001(0) | 0.003(0.01) |
| $CD15^+$Siglec-$8^+$%(%) | 0.038(0.05) | 0.015(0.03) | 0.043(0.06) |
| $CD15^+CD193^+$%(%) | 0.019(0.03) | 0.012(0.01) | 0.023(0.05) |
| $CD15^+CD125^+$%(%) | 0.009(0.01) | 0.009(0.01) | 0.009(0.02) |

Notes: CD15- Siglec-8+%: CD15- Siglec-8+ proportion of granulocyte; $CD15^-CD193^+$%: $CD15^-CD193^+$ proportion of granulocyte; $CD15^-CD125^+$%: $CD15^-CD125^+$ proportion of granulocyte; $CD15^+$Siglec-$8^+$%: $CD15^+$Siglec-$8^+$ proportion of granulocyte; $CD15^+CD193^+$%: $CD15^+CD193^+$ proportion of granulocyte; $CD15^+CD125^+$%: $CD15^+CD125^+$ proportion of granulocyte; IQR: interquartile range.

Table S5 Peripheral blood cytokines in patients with COVID-19 according to disease outcome

| | Total n=32 | Non-deceased n=25 | Deceased n=7 | *P* value |
|---|---|---|---|---|
| IL5(median, IQR, pg/ml) | 1.00(3.30) | 0.91(5) | 1.75(1.47) | 0.503 |
| IL13(median, IQR, pg/ml) | 4.00(14.71) | 5.42(13.38) | 2.58(23.79) | 0.929 |
| IL2(median, IQR, pg/ml) | 0(2.54) | 0(1.63) | 0(4.50) | 0.755 |
| IL6(median, IQR, pg/ml) | 6.11(29.24) | 5.62(23.02) | 6.48(66.08) | 0.447 |
| IL9(median, IQR, pg/ml) | 4.02(9.58) | 2.08(8.26) | 6.71(9.40) | 0.207 |
| IL10(median, IQR, pg/ml) | 5.79(8.01) | 5.81(7.93) | 5.03(8.76) | 0.894 |
| INFγ(median, IQR, pg/ml) | 15.31(38.28) | 14.30(37.54) | 25.99(69.29) | 0.859 |
| TNFα(median, IQR, pg/ml) | 0.13(22.07) | 0(21.33) | 14.96(82.48) | 0.148 |
| IL17A(median, IQR, pg/ml) | 0.04(2.99) | 0(2.69) | 1.56(3.93) | 0.370 |

| | | | | |
|---|---|---|---|---|
| IL17F(median, IQR, pg/ml) | 0(1.99) | 0(2.52) | 0(0.21) | 0.447 |
| IL4(median, IQR, pg/ml) | 2.07(3.33) | 2.18(3.78) | 1.96(3.27) | 0.859 |
| IL22(median, IQR, pg/ml) | 2.39(8.02) | 2.40(6.85) | 2.38(15.11) | 0.624 |

Notes: Data did not conform to normal distribution and were analyzed using the Mann–Whitney U-test, $P < 0.05$ is considered significant.
